# The Enigma of Centriole Loss in the 1182-4 Cell Line

**DOI:** 10.3390/cells9051300

**Published:** 2020-05-23

**Authors:** Alain Debec, Benjamin Loppin, Chunfeng Zheng, Xiuwen Liu, Timothy L. Megraw

**Affiliations:** 1 Institute of Ecology and Environmental Sciences, iEES, Sorbonne University, UPEC, CNRS, IRD, INRA, F-75005 Paris, France; 2 Laboratoire de Biologie et de Modélisation de la Cellule—CNRS UMR 5239, École Normale Supérieure de Lyon, University of Lyon, F-69007 Lyon, France; 3 Department of Biomedical Sciences, College of Medicine, Florida State University, Tallahassee, FL 32306-4300, USA; chunfeng.zheng@med.fsu.edu; 4 Department of Computer Science, Florida State University, Tallahassee, FL 32306-4530, USA; liux@cs.fsu.edu

**Keywords:** centriole, centrosome, *maternal haploid*, *Drosophila melanogaster*, cell line, haploid cells

## Abstract

The *Drosophila* *melanogaster* cell line 1182-4, which constitutively lacks centrioles, was established many years ago from haploid embryos laid by females homozygous for the *maternal haploid (mh)* mutation. This was the first clear example of animal cells regularly dividing in the absence of this organelle. However, the cause of the acentriolar nature of the 1182-4 cell line remained unclear and could not be clearly assigned to a particular genetic event. Here, we detail historically the longstanding mystery of the lack of centrioles in this *Drosophila* cell line. Recent advances, such as the characterization of the *mh* gene and the genomic analysis of 1182-4 cells, allow now a better understanding of the physiology of these cells. By combining these new data, we propose three reasonable hypotheses of the genesis of this remarkable phenotype.

## 1. Introduction

The centriole has likely been the subject of more controversy among cell biologists than any other organelle. As the structural core of the centrosome, the centriole was originally considered to be the “organ for cell division” by Van Beneden and Boveri, who discovered and investigated the centrosome in the 1880s [1]. Current consensus has established that animal cells can divide without centrioles, although the fidelity of chromosome segregation is impaired. Nevertheless, some organisms accomplish acentrosomal cell division as the normal course of development [1,2,3,4,5,6].

Despite recent advances in defining centriole biogenesis and the roles of centrioles in cell physiology [7,8,9,10,11,12,13,14], many questions still remain. In general, biologists aim to understand these issues concerning the centriole: (1) Why are centrioles and centrosomes necessary when mitosis can be accomplished without them? (2) How is the biogenesis of the centriole controlled?

The first example of an established animal cell line constitutively lacking centrioles was the 1182-4 *Drosophila* cell line. Although this has been firmly established by two extensive ultrastructural studies [15,16], the origin of this remarkable phenotype has remained elusive for many years. Recently, however, the *1182* mutated gene (renamed “*maternal haploid* (*mh*)”) has been characterized [17,18]. This allows us to re-examine this “cold case” and re-start the work where it left off. In this study, we review our current understanding of the maternal haploid “saga” from a historical perspective to explore the topic, connecting the original findings on the 1182-4 cell line with our understandings of the *mh* mutant that initiated the investigation. We bring new data concerning the genomic analysis of the 1182-4 cells. We conclude by discussing the possible explanations of the intriguing disappearance of centrioles in this cell line and aim to provide more clues to solve this longstanding mystery.

## 2. Material and Methods

### 2.1. Genomic DNA Preparation

*1182-4* cells from one confluent 100 mm plate were harvested and centrifuged at 2000 RPM for 2 min in 15 mL conical tubes. The cells were washed (resuspended and centrifuged) twice in PBS. The cell pellet was digested in a 500 μL digestion buffer (100 mM NaCl, 10 mM TrisCl, pH 8, 25 mM EDTA, pH 8, 0.5% SDS, 0.1 mg/mL Proteinase K) for 2 h at 50 °C. The sample was subsequently extracted with 500 μL Phenol/CHCl_3_/Isoamylalcohol 2 times and 500 μL CHCl_3_ once, the aqueous phase adjusted to 0.3 M sodium acetate with a pH of 5.2, and precipitated with 2.5 volume of 95% ethanol. The genomic DNA pellet was then washed with 70% ethanol, air dried, and dissolved in 200 μL of TE (10 mM Tris pH 7.5, 1 mM EDTA) buffer. RNAase was added to remove the residual RNA, followed by a phenol/CHCL_3_ extraction and ethanol precipitation, as above.

### 2.2. mh Expression Construct

The coding sequence for *mh* was amplified by PCR from plasmid pW8-attB-mh-V5 [17] and cloned into pENTR™/D-TOPO and then recombined into the pMT-Dest48 vector through a Gateway LR recombination using the manufacturer’s protocol (Invitrogen, Carlsbad, CA, USA). The resulting plasmid (pMT-mhV5) was transfected into *1182-4* cells with Lipofectamine 2000 using the manufacturer’s protocol (Invitrogen, Carlsbad, CA, USA). The cells were fixed and stained using established protocols [19].

## 3. Historical Perspective: The Origins of the Acentriolar 1182-4 Drosophila Cell Line

The *1182* mutation was first isolated from an EMS mutagenesis screen for X chromosome genes involved in female fertility [20,21]. For this class of mutation, the homozygous females are viable with a normal phenotype, except that they are sterile. They either present no fecundity (no eggs laid), or they produce eggs that are unable to develop or develop into embryos that do not hatch (maternal effect embryonic lethality). Among these 95 isolated X-linked female sterile mutants (*fs (1)Audit*) [20,21]), seven produced haploid embryos [21]. For these seven mutants, embryo development arrests either in early (preblastoderm) or late (organogenesis) stages. One of them, the mutant 1182^ts^, was chosen as advancing the farthest in embryonic development, although no larvae emerged. Initially, the 1182 mutant was thermosensitive with a normal development at 18 °C, but over time it began to drift towards a mutant stock fully penetrant at any temperature. The gene associated with this new mutant was renamed *maternal haploid (mh)* as it was soon clear that the Y chromosome was always absent in the haploid chromosomes set, even though the spermatozoon penetrates the egg, and the *fs(1)1182* mutant was designated *mh^1^* [22]. Loppin et al. [23] then provided a more detailed study of the early events of fertilization in the *mh* mutant, establishing that paternal chromosomes are lost at the first zygotic mitosis (see part 4).

With the goal to establish haploid cell lines of *Drosophila melanogaster*, many primary cultures were initiated from dissociated embryos derived from homozygous *mh^1^* mothers. Finally, six immortalized cell lines were obtained [24]. The karyotype evolution of these lines was followed for the first few months of cultures [25]. At first, all the lines showed a high proportion of haploid cells (80–100%), but most of them spontaneously diploidized and lost the haploid cells after 6–24 months. However, one line named 1182-4 was found to be stable, retaining a high proportion (80–90%) of haploid cells over years of culture and was selected for future experiments. Primary cell cultures from the embryos produced from crosses between *mh^1^*/*mh^1^* females with males bearing a ring X chromosome not only confirmed the maternal origin of the haploid genome but also demonstrated that all diploid cells arise from pre-existing haploid cells, as all of them presented two rod-shaped X chromosomes and never a ring-shaped one.

The presence of numerous dikaryons in the culture suggests a mechanism for the formation of isogenic diploid cells—a lack of cytokinesis followed later by a fusion of the nuclei of two sister cells, This hypothesis was demonstrated many years later, as it was shown that the centriole is involved in cytokinesis [26]. In addition, the detailed analysis of karyotypes in the 1182-4 cell line shows a surprising occurrence (up to 0.5%) of aneuploid constitutions with monosomies for the 2nd or the 3rd chromosomes; such monosomies are lethal for the flies. It is unknown if such constitutions are also cell lethal, but they have never been observed in other *Drosophila* cell lines [27].

These data suggested a cell division impairment and led us to suspect a mitotic defect in these cells. Using light microscopy, we examined the mitotic spindles in 1182-4 cells and found that they were frequently barrel-shaped, a classical characteristic of acentriolar mitosis. The first straightforward idea (but see part 6) was that, together with the defective paternally-transmitted chromosomes, the sperm basal body could also be lost, resulting in embryos developing with acentriolar spindles [15,16]. This possibility was also supported by the finding that the early cleavages of mouse embryos occur without centrioles [28]. Even if this situation occurs only transiently in the mouse embryo, this was a strong argument that mitosis can be accomplished without centrioles in animal cells. It was generally believed that centrosomes were essential for mitosis, but this assumption had not been formally tested. These considerations led us to undertake an ultrastructural observation of these cells to investigate the hypothesis of a loss of centrioles in the 1182-4 cell line.

During the 1980s, specific antibodies against *Drosophila* centrosome constituents were unavailable and a thorough EM study was the only approach possible to observe fly centrioles. Examinations of 4000 random cell profiles plus the serial reconstructions of mitotic cells with no detection of centrioles led to the conclusion that centrioles were absent in the 1182-4 cell line [15]. A diploidized subline of 1182-4 was also studied with the same conclusion, demonstrating that the acentriolar state is not strictly linked to the haploid constitution of these cells (see also part 6).

As demonstrating the absence of centrioles poses difficulties and is subject to controversy, a second study was also undertaken using high voltage electron microscopy. This allowed the screening of whole cells by 1µm-thick serial sectioning. Again, the conclusion held that 1182-4 cells are free of centrioles and therefore can divide without them [16]. These revelations that animal cells can divide continuously challenged long-held views on the mechanics of mitosis, yet were not widely appreciated until work in vertebrate cells and in *Drosophila* in vivo confirmed that an alternative acentrosomal mode of spindle assembly and mitosis can occur widely and efficiently. Nevertheless, it is important to point out that while the loss of centrioles with *sas-4* mutants [29] and the loss of functional pericentriolar material (PCM) with *centrosomin* (*cnn*) mutants [30] produce adult flies, they are severely impaired and lack all sensory detection when centrioles are absent due to their lack of cilia; they are also maternal effect lethal and male sterile when PCM is impaired.

In the following years until the molecular identification of *mh* [17], different studies addressed how 1182-4 cells accomplish their mitosis, but there were no more advances in the nature of the *mh* gene or the basis of centriole loss in the 1182-4 cell line. Nevertheless, the study of the early embryogenesis of *mh* embryos was informative in many aspects. It demonstrated that early syncytial divisions are controlled by the nucleo-cytoplasmic ratio, as haploid embryos undergo an extra syncytial division (15 cycles instead of 14) [31]. In addition, it revealed that the expression of pair-rule segmentation genes like *fushi tarazu* is independent of cellular density [32].

The progressive availability of antibody probes against centriolar components and the advent of modern light microscopy tools gradually permitted a more detailed analysis of acentriolar mitosis. In particular, using antibodies raised against *Drosophila* gamma tubulin, it was demonstrated that centrioles participate in the focal concentration of gamma tubulin. In absence of centrioles, a localized concentration of gamma tubulin can be formed at the poles of the mitotic spindles, but these structures are unstable or inconsistent [33]. Other centrosome/centriolar proteins have also been examined in the 1182-4 cell line, such as Cnn, D-PLP, and DSas-4 [34]. These proteins are usually dispersed or only form small aggregates at the spindle poles, consistent with a lack of centrioles, which are required to anchor the PCM. Together with the results of the previous study [33], these data indicated that centrioles are essential for the spatial organization of the mitotic poles. The formation of acentriolar microtubule-organizing centers in the 1182-4 cell line was subsequently studied more in-depth [34].

To summarize, the very existence of the 1182-4 cell line established a defining role for the centriole in organizing microtubules in somatic animal cells, and in particular during mitosis. The ability of cells to organize a mitotic spindle and execute mitosis without centrioles has been confirmed in mammalian cells and in vivo in *Drosophila* [1,29,35,36,37]. However, the origin of the acentriolar state of 1182-4 cells remains elusive, but we provide some speculation based upon the evidence so far (see part 6).

## 4. Rebound: Identification of the *mh* Gene

The characterization of another «gynohaploid» *fs(1)* mutant named *sésame* (*ssm*) [38] reactivated the interest in *mh* in 2000. Complementation tests revealed that *ssm* and *mh* affected different genes, which was also evident from cytological analyses of fertilization. In eggs from *ssm* females, the male pronucleus remains abnormally small and condensed and is, in most cases, excluded from the first zygotic spindle [38,39]. In *mh* eggs, pronuclear formation appears normal but paternal chromosomes fail to properly recondense in the metaphase of the first mitosis. As a consequence, paternal chromatin forms a chromatin bridge in anaphase and telophase which eventually breaks, resulting in the random segregation of the paternal chromatin mass between the two sister nuclei [23]. About 20% of *mh* embryos escape this first catastrophic mitosis and develop as gynohaploids. The first gonomeric spindle in *mh* eggs appears normal, with two microtubule asters containing the centrosomal protein CP190. In many *mh* embryos, however, the first mitosis generates aneuploid nuclei occasionally associated with monoastral or anastral spindles [23]. Gynohaploid *mh* embryos that escape this early developmental arrest and are observed at the syncytial blastoderm stage contain a vast majority of bi-astral spindles. Thus, maternal effect *mh* mutant embryos may have centriole biogenesis defects in the early zygote but do not have a block in centriole duplication if the early arrest is overcome.

Early attempts to molecularly map the *mh* gene (positioned between the *v* and *f* markers) were hampered by the lack of deficiency chromosomes for this region [23]. Eventually, the availability of a molecularly mapped non-complementing deficiency allowed the identification of a strong candidate gene (*CG9203*). This candidate was validated as the *mh* gene with the isolation of a second *mh* mutant allele and rescue experiments with genomic transgenes [17].

The *mh*/*CG9203* gene encodes the *Drosophila* ortholog of human *Spartan/DVC1*, a conserved metalloprotease originally characterized for its presumed role in translesion synthesis. More recently, Spartan was shown to be required for the processing of DNA-protein crosslinks (DPCs), a class of highly deleterious DNA damage caused by the covalent attachment of a variety of proteins to DNA bases. Work in human cells, yeast, nematodes, and flies have established that Spartan is required to remove protein adducts on DNA that can otherwise block the passage of the DNA replication complex [40]. Mutations in the human *SPRTN* gene cause Ruijs-Aalfs syndrome, a rare disease characterized by premature aging and cancer [41].

Surprisingly, sequencing the *CG9203* gene from *mh^1^* flies revealed the presence of five non-synonymous substitutions, including one that affects a highly conserved residue in the SprT domain (M151K) and likely inactivates protease activity [17]. The independent identification of *mh* by another group confirmed the presence of five point mutations in the *mh^1^* stock, including the one in the SprT domain [42]. The origin of these additional SNPs in the *mh^1^* allele is unclear, but their identification in the 1182 haploid cell line (see below) suggests that they were already present at the time of the mutagenesis or that they appeared shortly after. Intriguingly, *mh* was identified as one of the DNA repair genes with a significant adaptative natural variation to UVB light exposure [43], opening the possibility that these SNPs could indeed be natural variants.

Recently, Puah et al. found that haploid nuclei in *mh* embryos occasionally fuse during cleavage mitoses, giving rise to 2n, 3n, or 4n nuclei through a process named nuclear collisions [42]. Nuclear cleavage divisions in the *Drosophila* early embryo occur in a syncytium, where proper division relies on astral microtubules from centrosomes to provide spacing between nuclei and prevent nuclear collisions and consequent aneuploidy [44]. Thus, the nuclear collisions seen in *mh* mutant embryos are reminiscent of the nuclear fusions observed in *centrosomin*-deficient embryos [45], suggesting that *mh* could also affect centrosome function [42]. However, since other gynohaploid mutants were not examined in this study, it is not clear whether these nuclear fusions are just a consequence of haploid development. Although it is currently difficult to directly connect the molecular role of Spartan in DNA damage repair with the centrosome function, it is known that DNA replication stress in early *Drosophila* embryos induces centrosome inactivation [46]. This safeguarding mechanism allows the elimination of aneuploid or incompletely replicated nuclei from the developing embryo [47]. Recent work shows that mild replication stress causes premature centriole disengagement in early mitosis, [48] which could subsequently lead to centriole loss during division.

**Figure 1:**
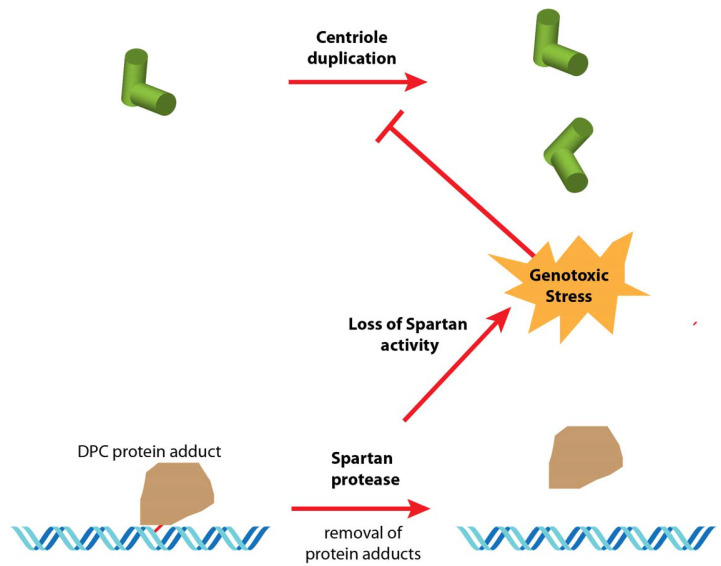
Hypothesis 1: loss of Spartan activity impacts centriole duplication in cell culture. In wild-type cells, the Spartan metalloprotease removes covalent DNA protein crosslinks (DPC), which can block the passage of the DNA replication complex. In *mh^1^* mutant cells, the lack of Spartan activity leads to the persistence of DPCs, possibly resulting in chronic DNA replication stress in these constantly dividing cells. This, in turn, induces a DNA damage response that could affect either the centriole duplication cycle or even the centriole disassembly.

Although the essential function of Spartan in *Drosophila* is to maintain DNA integrity in the male pronucleus, Spartan is likely required throughout development [17,18,42]. It is thus conceivable that the loss of centrosomes in the 1182 cell line could be related to chronic DNA replication stress caused by the lack of Spartan activity (Hypothesis 1, Figure 1; see part 6).

## 5. Genomic Analyses of 1182-4 Cells

The genetic basis of centriole loss in the 1182-4 cell line remains mysterious. The cell line was derived from the *mh^1^* mutant, but the *mh^1^* mutant does not show a loss of centrioles in vivo in third instar larval brains or in maternal effect embryos. One possibility is that the cell type that the 1182-4 cell line is derived from is susceptible to centriole loss when Spartan activity is impaired, whereas the other cell types that we examined are not. First, we sequenced the *mh* from genomic DNA prepared from the 1182-4 cell line and confirmed the allelic identity with the *mh^1^* mutant. As mentioned in the previous section, the 1182-4 cell line was verified to come from the *mh^1^* mutant stock, as the *mh^1^* mutant allele is identical in sequence, containing not only the responsible mutation in the SprT domain (M151K) but also four additional polymorphisms that differed from the Flybase reference genome sequence [17]. Thus, the *mh^1^* mutation is associated with centriole loss in the 1182-4 cell line, but it is not clear that it is causative.

This led us to examine the genome of the 1182-4 cell line for mutations in the essential centriole biogenesis genes. We examined the sequences of the core proteins involved in centriole biogenesis listed in Table 1, using the available sequence data for 1182-4 from the ModENCODE project [49]. There were notable differences but no clear evidence that any of the genes had loss of function mutations to explain the loss of centrioles in the cell line. The *ana1* gene, which is highly divergent among species and is the ortholog of mammalian *CEP295* [50], had several sequence differences in the open reading frame (ORF) relative to the reference genomic sequence, but none were predicted to create nonsense or frameshift mutations and are likely to be natural variants.

To test the idea that *mh* is causative of centriole loss in the 1182-4 cell line, we transfected the cells with a plasmid that expresses Spartan. In transfected cells that showed Spartan expression (by a C-terminal V5 tag), there was no restoration of centrioles/centrosomes in the cells. Given that the experiment was limited to a short timeframe and the unknown kinetics of centriole restoration in this context, we hesitate to conclude that *mh* is not responsible for the phenotype.

## 6. Hypotheses and Perspectives

Our initial hypothesis (see part 3)—the loss of centriole/basal body brought in by the spermatozoon—is contradicted by the fact that haploid embryos issued from *mh^1^/mh^1^* mothers are able to accomplish their syncytial mitosis. It is well established that centrioles are essential for early syncytial divisions. For example, embryos arising from germline clones devoid of the Sas-4 or SAK/PLK4 functional proteins halt very early after some few mitoses [44,51,52]. In addition, the *mh* haploid embryos present normal centrosome asters during their early development [23]. Therefore, it is unclear why the 1182-4 cell line, derived from *mh^1^* mutant embryos, loses centrioles when this does not occur in vivo.

An alternative hypothesis would be that some adaptive event occurred during the establishment of the 1182-4 cell line—for example, a mutation in an essential gene required for the maintenance of centrioles. Such an event could conceivably favor the multiplication of the mutated cells and be selected during the early period of primary culture. Interestingly, the 1182-4 line, despite its mitotic abnormalities (chromosome losses and cytokinesis defects), grows very rapidly. The genome analysis of 1182 cells (part 5) has not revealed any mutations in the “usual suspects” list of known genes devoted to centriole biogenesis (Table 1), although of course other genes involved in this pathway probably remain to be discovered.

The precise tissue origin of most *Drosophila* cell lines is usually unknown, at least for the lines established from whole dissociated embryos. We could then imagine alternatively that the 1182-4 cells originated from a tissue where centriole biogenesis is inactive. This also seems improbable, as no such acentriolar dividing tissue is known in *Drosophila*. Moreover, more than 150 *Drosophila* cell lines are listed in the Drosophila Genomics Resource Center (https://dgrc.bio.indiana.edu/cells/Catalog) and probably far more exist in many labs around the world, but there is no report of other spontaneous acentriolar *Drosophila* cell lines. Only some of these are acentriolar because they have been specially established from *Sas-4* mutant embryos [53].

The examination of the 1182-4 cell line by immunofluorescence against CP190, a centrosome marker which localizes to centrosomes at mitosis [54], demonstrated an absence of centrosomal signal in 100% of the cells, and the opposite in control Kc cells [55]. The CP190 protein is still present in 1182-4 cells, but this protein is not gathered around centrioles. Interestingly, a survey with the same technique of four other cell lines derived from *mh* mutant embryos revealed the presence in each line of a portion of acentrosomal cells (from 30% to 90%). Such cells are likely acentriolar (although not followed by EM, an impossible task for mixed cell populations). This demonstrates that these two hypotheses (exceptional mutational event and peculiar tissue origin) can be ruled out. In other words, the lack of centrioles in 1182-4 cells is not adaptive but linked to their special origin.

To determine the basis of centriole loss in the 1182-4 cell line, we evaluated the peculiarities of this cell line.

The first idea that came to mind was the fact that these cells are mutant for the *mh* gene. It is striking (and satisfying) that, after so many years after the establishment of the line, the same mutation and associated polymorphisms in the 1182-4 cells persist in the present day *mh^1^* allele. The first hypothesis (Hypothesis 1, Figure 1) to explain the loss of centrioles in the 1182-4 cell line is thus directly linked to the function of Spartan in processing DPCs. In this case, the presence of unrepaired DPCs could cause a replication stress for Spartan-deficient cells. Such a possibility could be experimentally tested by an alternative means to impair the DNA damage response, such as by the inactivation of the checkpoint kinase Chk2. Alternatively, it could be interesting to establish cell lines from other alleles of *maternal haploid*. It should be emphasized, however, that such a direct implication of *mh^1^* in centriole loss could only be imagined as a long-term effect during cell culture. Thus, the *mh^1^/mh^1^* flies are perfectly viable and normal except for their sterility and they do not present the uncoordinated behavior and early death phenotype associated with centriolar defects such as seen with acentriolar *Sas-4* mutant flies [29].

Another consideration is that the 1182-4 cell line has an impairment, possibly caused by *mh^1^* itself, required for de novo centriole biogenesis. If Spartan is required for this, it might not be evident in vivo, where the centriole duplication fidelity is high. This is a testable idea and would suggest that de novo centriole biogenesis is more tightly linked to the DNA damage pathways than templated centriole duplication, for which many links have already been made. We must also keep in mind that the re-introduction of the WT allele of the *mh* gene does not lead to acute centriole recovery (see part 5).

Although the *mh^1^* mutation could not be directly (structurally) responsible for the acentriolar state of 1182-4 cells, we can also consider this peculiarity as a consequence of the developmental perturbation created by this mutation.

The immediate consequence of *mh^1^* mutation is the loss of paternal chromosomes, which leads to a gynohaploid development. A recent work has demonstrated that frequent centrosome and centriole losses occur in the near-haploid HAP1 human cell line and in haploid mouse parthenogenetic embryos [56]. There is also a correlation between the over-duplication of centrioles and ploidy in tetraploid cells. The authors conclude that the lack of coordination between centrosome duplication and chromosome replication cycles contributes to the loss of centrioles in haploid cells. It is noteworthy that a normal centriole duplication cycle occurs in naturally haploid cells such as in males of Hymenoptera. Even microtubule-less centrioles in the haploid early male larvae of wasps are duplicated identically to centrioles in diploid somatic cells [57]. However, we can imagine that such haploid constitution in Hymenopteran males has been selected throughout evolution; this can be considered as a physiologically normal condition, in contrast to most other organisms where haploidy might be incompatible with the fidelity of centriole duplication.

**Figure 2:**
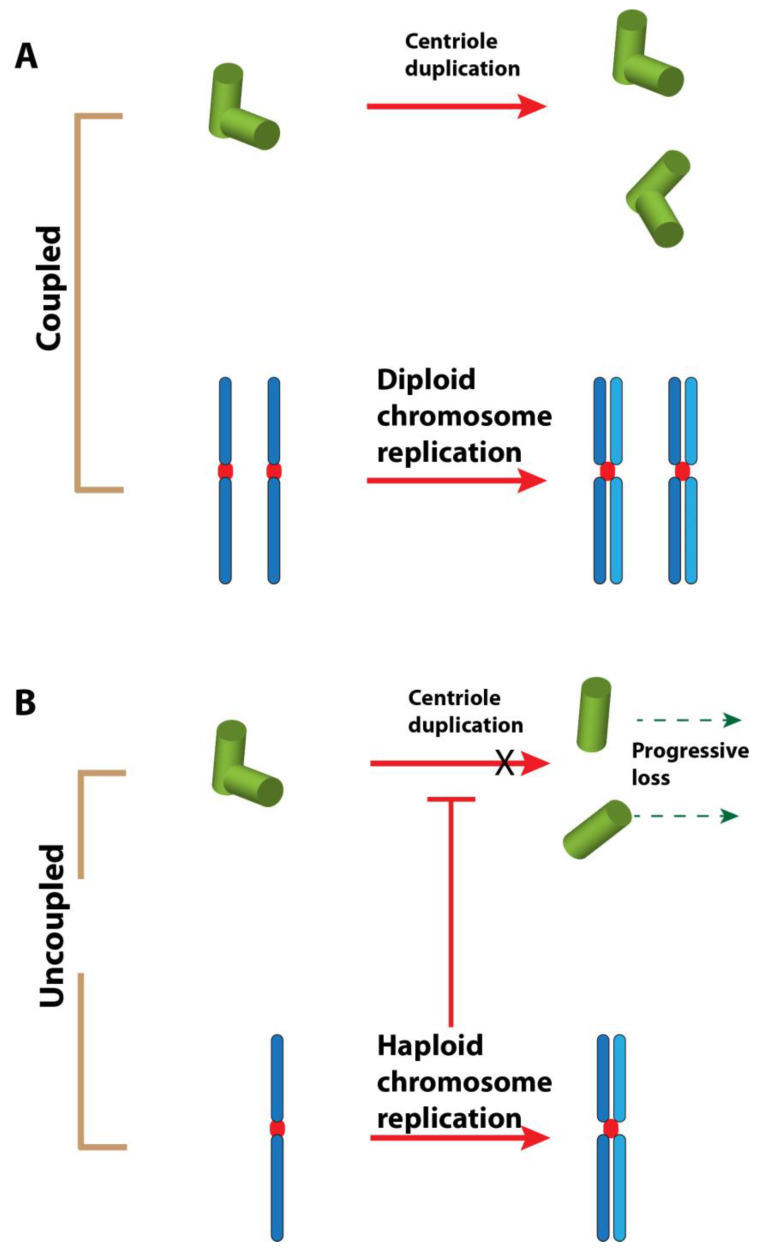
Hypothesis 2: haploidy causes centriole loss. (**A**). In a diploid cell, the chromosome replication and centriole duplication cycles are tightly coordinated during the cell cycle. (**B**). In a haploid cell, these events are uncoupled. The result could be an incomplete centriole duplication cycle. The licensing step for the duplication of the centriole could also be affected [54]. The repetition of uncoordinated events could lead to the loss of centrioles after some cell cycles.

This relationship between ploidy and centrosome cycle certainly represents an appropriate argument to explain the centriole loss in 1182-4 cells—this is Hypothesis 2 (Figure 2). However, in Drosophila 1182-4 cells, the return to diploidy does not restore any centriole formation as the diploid subline derived from it (1182-4D) is also devoid of centrioles. In the near-haploid HAP1 human cell line, only a fraction of the cell population is acentriolar. We could surmise that centriole loss is effectively due to an uncoordinated centrosome cycle in haploid cells, but for an unknown reason the return to a diploid state is not followed by the restoration of centrioles. It is also clear that a quasi-normal development of a *Drosophila* adult fly can occur without centrioles [29], and therefore it is possible that the constraints of centriole duplication cycle are less stringent in *Drosophila* than in vertebrates. It would be highly interesting to establish new haploid cell lines from the other known mutants leading to haploid embryos—such as *sésame* [38], *deadhead* [58], or *ms(3)K81* [59]—and check their centriolar status. This would allow us to distinguish between Hypotheses 1 and 2.

**Figure 3:**
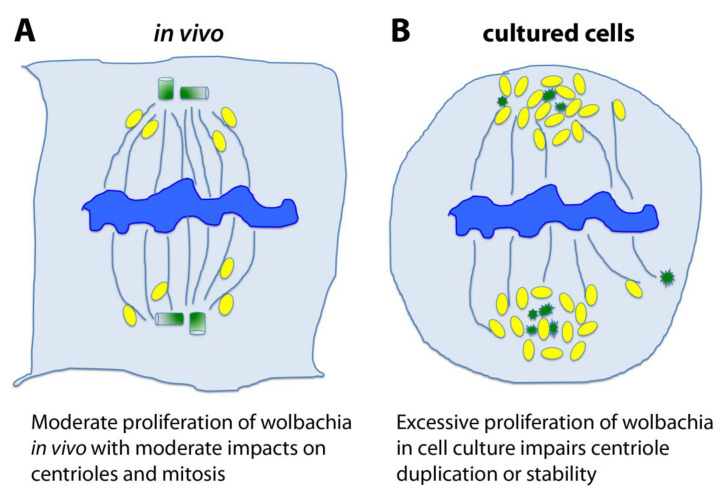
Hypothesis 3: chronic and excessive *Wolbachia* infection causes centriole loss in cell culture. (**A**). *Wolbachia* are intracellular bacteria that infect many *Drosophila* strains and, in particular, the original stock from which the *mh^1^* mutation was isolated. In physiological conditions, these bacteria depend on their cell host survival and their proliferation is moderate. The microbes associate with and traffic upon the microtubule (MT) cytoskeleton, and even if they associate with the poles of the mitotic spindle they do not typically cause centriole loss in vivo. (**B**). In cultured cell lines, *Wolbachia* seems to be out of control and tend to overproliferate. As many *Wolbachia* bacteria gather towards spindle poles, it is possible that their overwhelming presence leads to the disruption of the centrosome and centriole instability. In these schematics, the chromosomes are blue, the MTs are blue threads, centrioles or degraded centrioles are green, and *Wolbachia* are yellow ovals.

At last, there is also an alternative and intriguing Hypothesis 3 (Figure 3); this is totally outside the previous considerations, but it is one we cannot exclude—the effect of the endosymbiotic bacteria *Wolbachia pipientis*. In fact, the initial 1182-4 cells were infected by *Wolbachia* before they were cured by antibiotics [60]. These intracellular bacteria were simply derived from the initial fly stock bearing the *mh^1^* mutation, which itself was infected. *Wolbachia* are able to manipulate the early embryonic cell cycles of their host [61]. They indeed cause the Cytoplasmic Incompatibility (CI) phenomenon, which generally leads to embryonic death in crosses between infected males and uninfected females, helping to spread the bacteria in the fly population by selecting for infected females. There is a striking similarity between CI and *mh* phenotypes, which both lead to the early loss of paternal chromosomes in the zygote [62]. *Wolbachia* navigate along microtubules and localize to the spindle poles during mitosis. In cultured cells, *Wolbachia* often over-proliferate. Examination of mitoses in infected cells show frequent mitotic abnormalities, such as scattered spindles poles and monopolar spindles (Debec and Sullivan, unpublished observations). We could thus speculate, at least in cell culture conditions, that *Wolbachia* could affect some cytoskeleton structures including centrioles. This hypothesis is more speculative than the two previous ones (genotoxic stress or haploid constitution), but it would be worth investigating more carefully for the mitotic abnormalities in various *Wolbachia*-infected *Drosophila* cell lines.

## 7. Conclusions: Something Has Been Lost?

We cannot yet conclude at this stage what mechanism triggers centriole loss in the 1182-4 cell line, but it remains an intriguing question for which the answer is likely to reveal novel aspects of centriole biogenesis or maintenance. An attractive hypothesis is that the initial haploid state of these cells that could affect centriole homeostasis [56] (Hypothesis 2). We cannot preclude, however, the effect of genotoxic stress (Hypothesis 1) or the possible influence of *Wolbachia* (Hypothesis 3). However, one thing is striking: after reintroduction of wild-type mh expression, returning to the diploid karyotype, or eliminating *Wolbachia*, the 1182-4 cells do not recover centrioles.

We can speculate that the 1182-4 cell line has lost the capability for de novo centriole assembly. Normally, centriole duplication is tightly regulated and restricted to one duplication event per cell cycle [7,63]. However, when centrioles are depleted completely, either by antibody inhibition, laser or surgical ablation, genetic tricks such as the temporal depletion of SAK/Plk4, or the drug inhibition of Plk4, centrioles will assemble de novo [51,64,65,66,67,68]. This untemplated form of centriole biogenesis is slower and results in a range of centriole numbers that then proceed to replicate in the normal templated fashion. Many cell lines are poor at maintaining normal centriole numbers, and perhaps the 1182-4 cell line fails to replace them de novo when they are lost.

We are not offering an exhaustive set of hypotheses for why centrioles are lost in the 1182-4 cell line, but as we proceed to investigate this phenomenon further, we can explore two general categories of explanations. Either the centriole assembly pathway is blocked by an unknown mechanism, or an unidentified centriole maintenance pathway is inactive (or a destruction pathway is constitutively active). Related to the first category, it is possible that something has been impaired that is specifically required for the de novo pathway of centriole assembly, a process that remains very poorly understood. In this scenario, *mh^1^* mutant cells lose their centrioles so infrequently in vivo that that loss of de novo centriole assembly does not manifest itself, whereas the relatively high rate of centriole loss in cell culture shows this deficiency in the 1182-4 mutant cell line. If this hypothesis holds true, future work on the 1182-4 cell line could reveal important new insights into the de novo pathway of centriole biogenesis.

## Figures and Tables

**Table 1:** Genetic dispositions of centriole biogenesis genes in the 1182-4 cell line.

Gene	Aberrations *	Location of Aberration in Gene
*SAK*	1bp Del at 21509098	110 bp 5′ from transcription start.
*Sas-6*	None	
*Sas-4*	None	
*Asl*	None	
*Cep135/bld10*	1bp Ins at 15109935	5′ UTR
	2bp Del at 15112702	intron
	1bp Ins at 15112720	intron
	2bp Del at 15112725	intron
	1bp Ins at 15112962	intron
	20bp Del at 15115633	intron
	1bp Ins at 15119269	intron
*ana1*	1bp Del at 20356251	5′ UTR
	1bp Del at 20356417	5′ UTR
	3bp Del at 20359488	middle of ORF
	18bp Ins at 20359480	middle of ORF
	3bp Ins at 20361873 (CAG)	near 3′ end of ORF
*ana2*	24bp Del at 4786186	About 40 bp 3′ of transc stop.
*ana3*	None	
*Cp110*	1bp Ins at 21884283	5′UTR
	4bp Del at 21884285	5′UTR
	30bp Del before 21884288?	5′UTR
	2bp Ins at 21886886	intron
	2bp Del at 21886889	intron
	5bp Ins at 21886895	intron
	1bp Del at 21886898	intron
	4bp Del at 21888272	intron
	7bp Del at 21888275	intron
	36bp Del before 21888280?	intron
	3bp Ins at 21888375	intron
	14bp Del at 21888813	intron
	1bp Del at 21888879	intron

* Relative to Drosophila genome release 5 (Flybase)
